# Triple-negative breast cancer cells rely on kinase-independent functions of CDK8 to evade NK-cell-mediated tumor surveillance

**DOI:** 10.1038/s41419-021-04279-2

**Published:** 2021-10-23

**Authors:** Vanessa Maria Knab, Dagmar Gotthardt, Klara Klein, Reinhard Grausenburger, Gerwin Heller, Ingeborg Menzl, Daniela Prinz, Jana Trifinopoulos, Julia List, Daniela Fux, Agnieszka Witalisz-Siepracka, Veronika Sexl

**Affiliations:** 1 Institute of Pharmacology and Toxicology, University of Veterinary Medicine, Vienna, Austria; 2 Department of Medicine I, Medical University of Vienna, Vienna, Austria; 3 Comprehensive Cancer Center, Vienna, Austria

**Keywords:** Breast cancer, Immune evasion

## Abstract

Triple-negative breast cancer (TNBC) is an aggressive malignant disease that is responsible for approximately 15% of breast cancers. The standard of care relies on surgery and chemotherapy but the prognosis is poor and there is an urgent need for new therapeutic strategies. Recent in silico studies have revealed an inverse correlation between recurrence-free survival and the level of cyclin-dependent kinase 8 (CDK8) in breast cancer patients. CDK8 is known to have a role in natural killer (NK) cell cytotoxicity, but its function in TNBC progression and immune cell recognition or escape has not been investigated. We have used a murine model of orthotopic breast cancer to study the tumor-intrinsic role of CDK8 in TNBC. Knockdown of CDK8 in TNBC cells impairs tumor regrowth upon surgical removal and prevents metastasis. In the absence of CDK8, the epithelial-to-mesenchymal transition (EMT) is impaired and immune-mediated tumor-cell clearance is facilitated. CDK8 drives EMT in TNBC cells in a kinase-independent manner. In vivo experiments have confirmed that CDK8 is a crucial regulator of NK-cell-mediated immune evasion in TNBC. The studies also show that CDK8 is involved in regulating the checkpoint inhibitor programmed death-ligand 1 (PD-L1). The CDK8–PD-L1 axis is found in mouse and human TNBC cells, underlining the importance of CDK8-driven immune cell evasion in these highly aggressive breast cancer cells. Our data link CDK8 to PD-L1 expression and provide a rationale for investigating the possibility of CDK8-directed therapy for TNBC.

## Introduction

Triple-negative breast cancer (TNBC) cells lack the expression of estrogen and progesterone receptors, with normal human epidermal growth factor receptor type 2 (HER2) gene copy number and expression. TNBC accounts for approximately 15–20% of all breast cancers with high prevalence in young and African-American women. It is an aggressive disease with a high risk of relapse and mortality. Compared to other breast cancer subtypes, visceral metastasis formation is frequent and involves lung, bone, and brain [1–3]. Chemotherapy is the current standard-of-care treatment of TNBC in adjuvant, neoadjuvant, and metastatic settings. Although TNBCs are highly sensitive to chemotherapy, the frequent occurrence of relapse enforces the search and evaluation of novel therapeutic approaches [2].

In silico investigations of patient samples revealed an inverse correlation between recurrence-free survival and cyclin-dependent kinase 8 (CDK8) levels in various breast cancer types, however, a correlation separately for TNBC patients has not yet been reported [4–6]. CDK8 and its close homologue CDK19 are serine/threonine kinases and key players in transcriptional regulation. They form the kinase module of the Mediator complex, together with Cyclin C, MED12, and MED13 [7, 8]. The CDK8 submodule has as well been described to directly phosphorylate signaling molecules, including members of the Janus kinase and signal transducer and activator of transcription (JAK-STAT) pathway [9], transforming growth factor-β (TGF-β), and bone morphogenetic protein (BMP) receptor signaling [10]. By utilizing the JAK-STAT pathway, CDK8 can interfere and regulate immune responses [9]. A function of the CDK8-STAT1 axis was also reported in natural killer (NK) cells—showing that knockdown of CDK8 within NK cells enhances their cytotoxicity in vitro and in vivo by increasing the expression of lytic molecules [11]. Paradoxically, deletion of CDK8 in NK cells partially recapitulates the enhanced NK-cell-mediated cytotoxicity, but STAT1-S727 phosphorylation is unaltered in CDK8-deficient NK cells, presumably due to a compensatory function of CDK19 [12]. The use of a specific CDK8/CDK19 inhibitor showed supportive results that this effect is dependent on the kinase activity of CDK8 and the same study provides evidence that combination treatments with either anti-programmed death 1 (PD-1) monoclonal antibodies in a colorectal cancer model or SMAC mimetic in a breast cancer model increase the survival of mice [13].

In T cells, CDK8/CDK19 kinase inhibition induces the conversion of conventional T cells to regulatory T cells, enhancing antitumor surveillance and highlighting a role of CDK8/CDK19 in the repression of forkhead box protein P3 (FOXP3) [14].

Additionally, CDK8 has been identified as an oncogenic driver in a variety of cancer types including melanoma, colorectal cancer, and hematological malignancies [4, 6, 15, 16].

A recent study describes CDK8 as a potential therapeutic target in estrogen receptor (ER)-positive breast cancer cells, showing that estrogen-induced transcription depends on CDK8 kinase activity. Chemical inhibition of CDK8/CDK19 or genetic knockdown of CDK8 impaired cell growth and progression of this breast cancer type in vitro and in vivo [17].

Moreover, it has been shown that CDK8 contributes to epithelial-to-mesenchymal transition (EMT), a process which plays an important role in invasion and metastasis of breast cancer cells [18–20].

We here show that tumor-intrinsic CDK8 regulates invasiveness and metastatic capacity of TNBC cells and shields the tumor cells from NK-cell-mediated recognition and killing in a kinase-independent manner. These data suggest that CDK8 not only represses perforin and granzyme production, and thereby hampers NK-cell cytotoxicity, but also drives cancer-intrinsic immune evasion by regulating crucial immune checkpoints. We describe a novel CDK8–PD-L1 (programmed death-ligand 1) axis, which might provide a mechanistic explanation for the CDK8-driven NK-cell evasion and is of utmost therapeutic relevance as PD-L1/PD-1 blocking antibodies show promising results in clinical trials of TNBC patients. In summary, we identify CDK8 as an important regulator of tumorigenesis and describe it as a novel immune checkpoint in TNBC.

## Results

### CDK8 promotes regrowth of tumors and distant metastasis formation

**Fig. 1:**
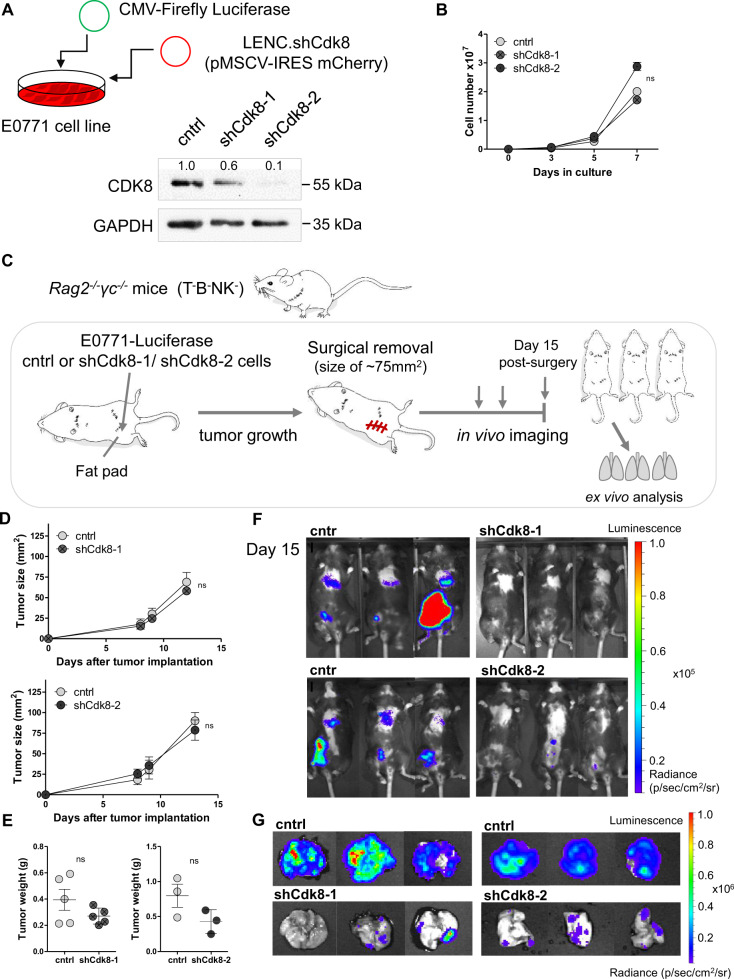
CDK8 supports the regrowth of tumors and distant metastasis formation. **A** TNBC E0771 cells were stably infected with a CMV-Firefly Luciferase construct and subsequently with two different CDK8-targeting shRNAs (shCdk8–1 and −2) or a control shRNA (cntrl), and the expression of CDK8 and GAPDH was analyzed by western blot. **B** Proliferation of E0771 cell lines infected with shCdk8–1, shCdk8–2 and the control cell line was assessed on day 3, 5, and 7 by flow cytometry. Symbols and error bars represent cell numbers in mean ± SEM of *n* = 3 technical replicates. **C** Experimental setup of experiments from (**D**–**G**) is shown. *Rag2*^*−/−*^*γc*^*−/−*^ mice were orthotopically transplanted with E0771 control, shCdk8–1, or shCdk8–2 cells (in independent experiments) and monitored for primary tumor growth. Tumors were surgically removed and regrowth and distant metastasis of tumor cells was monitored with in vivo imaging. **D** Primary tumor growth of control vs. shCdk8-1 or shCdk8-2 implanted mice, and **E** tumor weight of mice at operation. Symbols and error bars represent tumor area (mm^2^) in mean ± SEM. **F**, **G** Representative in vivo-imaging pictures of mice (**F**) and ex vivo lungs (**G**) of two independent experiments with either shCdk8-1 or shCdk8-2 compared to control, at day 15 post surgery (*n* = 3–5 mice per group).

To study the role of CDK8 in TNBC, we used shRNA-mediated knockdown of CDK8 in murine TNBC E0771 cells [21]. Immunoblotting confirmed the decreased CDK8 levels with two different hairpins to a varying extent (shCdk8–1 and shCdk8–2) (Fig. 1A). We did not detect any changes in proliferation (Fig. 1B), cell cycle distribution (Supplementary Fig. S1A), or survival upon CDK8 knockdown in vitro (Supplementary Fig. S1B).

To study primary tumor growth and metastasis formation in vivo, we used a well-established orthotopic breast cancer model [22] and transplanted Luciferase-expressing E0771 cells into the mammary fat pad of immunocompromised recombination activating gene 2 and interleukin 2 receptor gamma chain double knockout (*Rag2*^*−/−*^*yc*^*−/−*^) mice. When the primary tumor reached an area of 75 mm^2^, it was surgically removed and we followed disease progression by live imaging (Fig. 1C). In accordance with the unaltered cell proliferation in vitro, we did not observe any significant difference in primary tumor growth and weight irrespective of the expression of CDK8 (Fig. 1D, E). Persistent knockdown of CDK8 was confirmed in lysates from primary tumors (Supplementary Fig. S2A). CDK8-expressing cells gave rise to increasing Luciferase signals from day 9 to 15 post surgery at the site of injection, indicative of the regrowth of the tumor. In contrast, no Luciferase signals were detectable in mice that had received shCdk8–1 or shCdk8–2 knockdown cells (Fig. 1F and Supplementary Fig. S2B). At day 15 post surgery, the animals were sacrificed and analyzed for lung metastasis. Substantial metastatic spreading to the lungs had occurred upon injection with wild-type E0771 cells, while lung metastasis was significantly reduced in the absence of CDK8 (Fig. 1G and Supplementary Fig. S2C, D). This led us to conclude that tumor-intrinsic CDK8 promotes regrowth of tumors and distant metastasis formation in the orthotopic TNBC mouse model.

### Metastasis-related genes are deregulated upon CDK8 knockdown

**Fig. 2:**
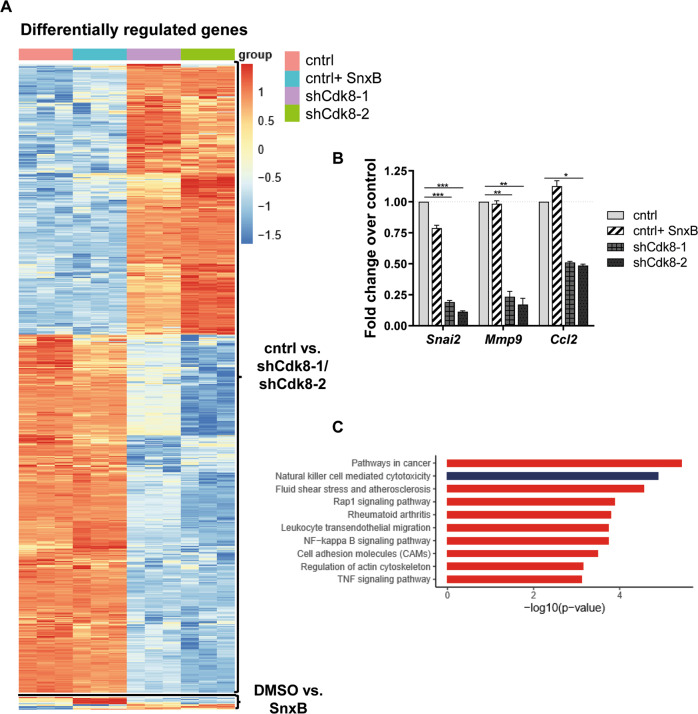
Loss of CDK8 regulates metastasis-related genes. **A** Heatmap of differentially expressed genes between untreated E0771 control or Senexin B (SnxB)-treated cells and shCdk8-1 or shCdk8-2 cell lines (RNA sequencing data; *n* = 3 replicates). **B** Real-time quantitative PCR of RNA extracted from the different E0771 cell lines. Fold change expression of *Snai2*, *Mmp9*, and *Ccl2* over E0771 control, normalized to *Rplp0*. One out of two to three independent experiments with similar outcome is depicted. Bar graphs and error bars represent mean ± SEM of technical triplicates. **C** Pathway analysis of 887 genes found to be differentially regulated by both CDK8-targeting shRNAs compared to control (overlap of cntrl vs. shCdk8-1 and cntrl vs. shCdk8-2) using KEGG pathway analysis.

To gain insights into how CDK8 promotes the metastatic potential of murine TNBC, we performed RNA sequencing (RNA-seq) and compared gene expression of E0771 control (cntrl), shCdk8–1, and shCdk8–2 cells. We included cntrl cells treated with the CDK8/CDK19 inhibitor Senexin B (SnxB) in the experimental setting. The efficiency of the SnxB treatment was confirmed by analyzing the CDK8 kinase substrate pSTAT1-S727 [9] by immunoblotting (Supplementary Fig. S3A). We identified 887 genes that were deregulated upon CDK8 knockdown by both shRNAs, whereas CDK8/CDK19 kinase inhibition by SnxB treatment resulted in only 25 deregulated genes (Fig. 2A and Supplementary Fig. S3B, C). Only nine genes were commonly deregulated by both CDK8 knockdowns and SnxB (Supplementary Fig. S3D). Differentially regulated genes between cntrl and shCdk8 cells included metastasis-related genes. We validated the downregulation of snail family transcriptional repressor 2 (*Snai2)*, matrix metalloproteinase-9 (*Mmp9)*, and chemokine (C-C motif) ligand 2 (*Ccl2*) in shCdk8 compared to cntrl cells, while expression of these genes was unaltered upon SnxB treatment (Fig. 2B). Besides qPCR in cell lines, we validated the altered expression of *Snai2* and *Mmp9* in cells derived from primary tumors (Supplementary Fig. S4A). Thus, we conclude that CDK8 is a crucial driver of metastasis in TNBC.

### CDK8 knockdown enhances NK-cell-mediated killing of TNBC cells in vitro

**Fig. 3:**
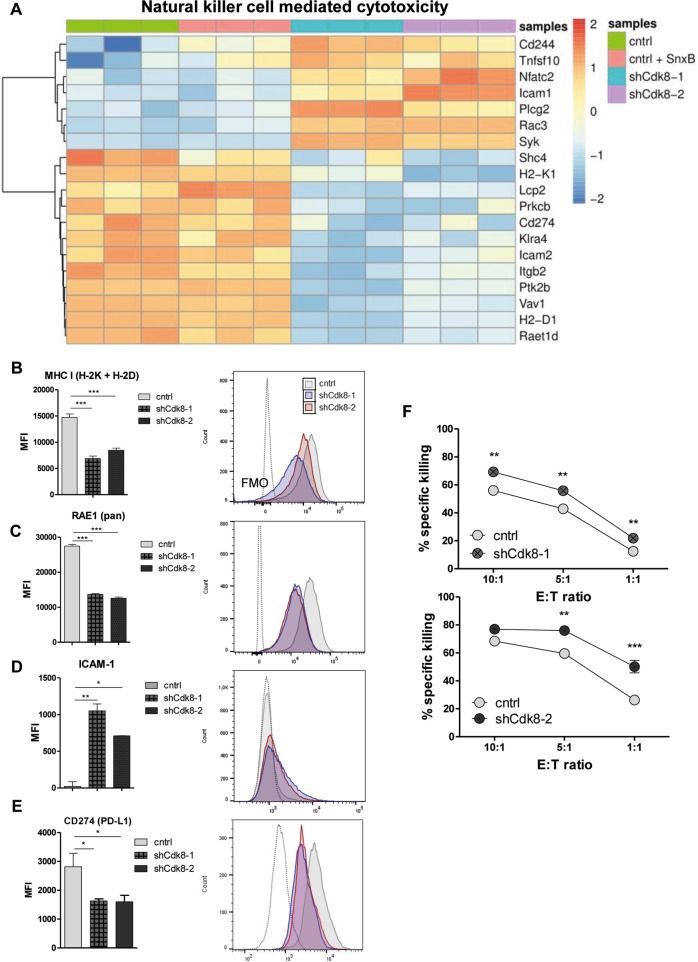
Loss of CDK8 enhances TNBC’s visibility to NK cells. **A** Heatmap of differentially expressed genes between untreated or Senexin B-treated E0771 control cells and shCdk8-1 or shCdk8-2 cell lines in the KEGG pathway “Natural killer cell-mediated cytotoxicity” (RNA sequencing data; *n* = 3 replicates). **B**–**E** Median fluorescence intensity (MFI) and representative histograms of (**B**) MHC I (H-2K + H-2D), (**C**) RAE1 (pan), (**D**) ICAM-1, and (**E**) CD274 (PD-L1) expression of E0771 control, shCdk8-1, and shCdk8-2 cells; expression levels of NK ligands were analyzed by flow cytometry. Bar graphs represent technical triplicates (mean ± SEM). **F** IL-2-expanded C57BL/6 NK cells were incubated for 4 h with CFSE-labeled E0771 control or shCdk8-1/shCdk8-2 tumor cells in effector:target (E:T) ratio of 10:1, 5:1, and 1:1. The specific killing was assessed by flow cytometry. One representative experiment out of two to three independent experiments is shown. Symbols and error bars represent mean ± SEM of technical duplicates.

We used KEGG to identify pathways deregulated by CDK8 knockdown, which revealed “Natural killer cell-mediated cytotoxicity” among the top hits (Fig. 2C). This included 19 deregulated genes (7 genes upregulated, 12 genes downregulated) upon CDK8 knockdown, which was not mimicked by CDK8/CDK19 kinase inhibition upon SnxB treatment (Fig. 3A). NK cells are innate lymphoid cells that have been implicated in preventing metastatic outgrowth [11, 23–25]. NK-cell activity is regulated by activating and inhibitory ligands on the surface of target cells [26, 27]. Self-major histocompatibility complex (MHC) class I molecules represent the most important inhibitory ligands that allow the discrimination of “self” and “missing self” [28]. CDK8 loss was associated with reduced expression of the MHC I genes H2-K1 and H2-D1 (Fig. 3A), which was validated by flow cytometry (Fig. 3B). We also confirmed the reduced protein levels of the activating natural killer group 2 member D (NKG2D) ligand ribonucleic acid export 1 (RAE1) and upregulation of the intercellular adhesion molecule 1 (ICAM-1) in CDK8-deficient cells by flow cytometry (Fig. 3C, D). No changes in MHC I and RAE1 expression were detected upon SnxB treatment (Supplementary Fig. S4B). Our transcriptome analysis of CDK8-deficient E0771 cells also revealed diminished expression of *Cd274*, encoding for PD-L1—a ligand of PD-1 and an important immune checkpoint protein. We confirmed the reduced expression of PD-L1 on protein level upon loss of CDK8 in E0771 cells (Fig. 3E).

These data point to CDK8 as a regulator of NK-cell-mediated recognition of TNBC cells. We thus performed in vitro cytotoxicity assays with IL-2-expanded splenic NK cells. E0771 cell lines were more susceptible to NK-cell-mediated cytotoxicity upon CDK8 knockdown (Fig. 3F), whereas no changes in NK-cell-mediated killing of E0771 cells were detected upon CDK8/CDK19 kinase inhibition, as observed by treating E0771 cell lines with SnxB for 48 h (Supplementary Fig. S4C). This led us to conclude that CDK8 in TNBC cells blocks NK-cell-mediated tumor recognition in a kinase-independent manner.

### Loss of CDK8 in TNBC increases NK-cell-mediated tumor surveillance in vivo

**Fig. 4:**
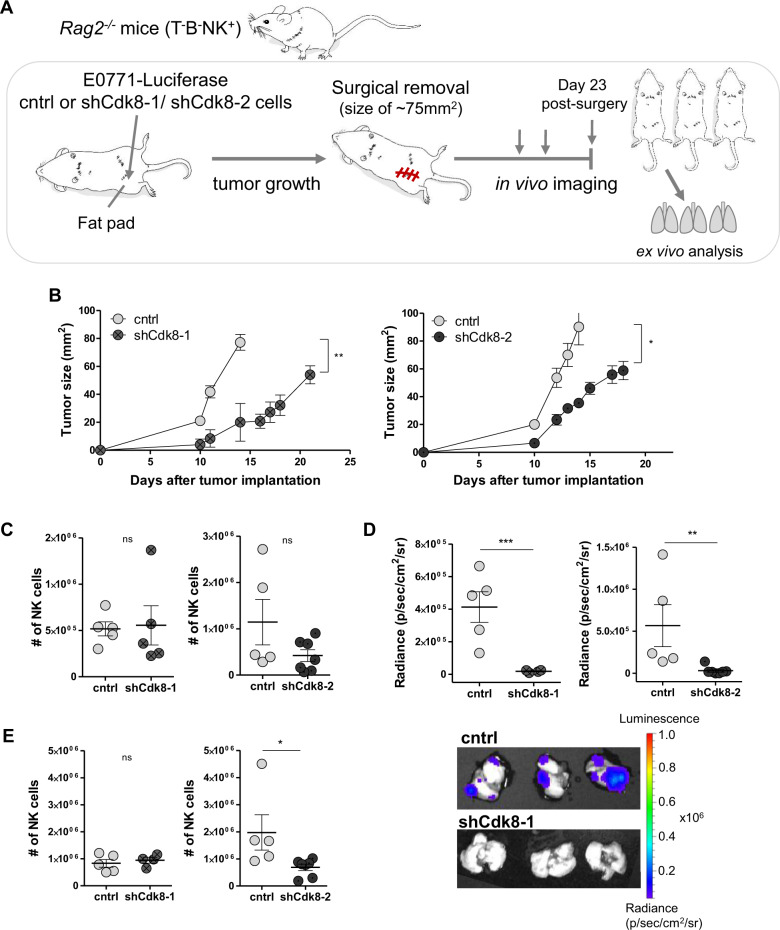
Loss of CDK8 in TNBC enhances NK-cell-mediated tumor surveillance. **A** Experimental setup of data shown in (**B**–**E**). *Rag2*^*−/−*^ mice were orthotopically transplanted with E0771 control, shCdk8-1, or shCdk8-2 cells (in independent experiments) and monitored for primary tumor growth. Tumors were surgically removed and regrowth and distant metastasis formation of tumor cells was monitored with in vivo imaging. **B** In vivo primary tumor growth for control vs. shCdk8-1 or shCdk8-2 E0771 cells in the left and right panel, respectively. **C** Total infiltrating NK-cell numbers (CD45^+^NK1.1^+^NKp46^+^) of digested primary tumors at day of surgery. **D** Radiance signals of lungs ex vivo measured by an in vivo-imaging system and representative pictures of lungs (lower panel) at the end of the experiment (day 23 post surgery). **E** Total numbers of infiltrating NK cells (CD45^+^NK1.1^+^NKp46^+^) of lung lysates were assessed. Symbols and error bars represent mean ± SEM (*n* = 4–9 mice per group).

To address the relevance of CDK8 in NK-cell-mediated recognition in an in vivo setting, we orthotopically injected CDK8-deficient and wild-type tumor cells into recombination activating gene 2 knockout (*Rag2*^*−/−*^) mice that rely on NK cells for tumor surveillance but lack T and B cells (Fig. 4A). Primary tumor growth was significantly delayed in mice transplanted with CDK8-knockdown cells (Fig. 4B), indicating an enhanced NK-cell-mediated tumor surveillance of the primary tumor. Surgical removal of the primary tumor was consistently performed at a tumor size of 75 mm^2^ (Fig. 4A), and the persistent knockdown of CDK8 was confirmed (Supplementary Fig. S5A). Tumor weight (Supplementary Fig. S5B) and the numbers of tumor-infiltrating NK cells (Fig. 4C) were comparable at the day of operation. In contrast to *Rag2*^*−/−*^*yc*^*−/−*^ mice, no re-growing of the tumors post-surgery at the site of injection was observed in *Rag2*^*−/−*^ mice (Supplementary Fig. S5C).

At day 23 post surgery, all mice were imaged, sacrificed, and analyzed for lung metastasis (Fig. 4A). In line with a role of NK cells in recognizing TNBC cells, radiance signals as a readout for lung metastasis were generally lower in *Rag2*^*−/−*^ compared to *Rag2*^*−/−*^*yc*^*−/−*^ mice (Supplementary Fig. S5D). These data confirm that NK cells are able to recognize and eliminate the metastatic spread of TNBC cells. The knockdown of CDK8 enhanced this effect; lung metastasis in *Rag2*^*−/−*^ mice were almost absent upon transplantation with CDK8-knockdown tumors (Fig. 4D). The numbers of infiltrating NK cells were comparable between lungs of shCdk8–1 knockdown and control transplanted mice; however, there was a significant decrease in NK-cell numbers in the lungs of mice transplanted with shCdk8–2 knockdown cells (Fig. 4E). In line with the altered metastasis formation, we found that the lung weights were elevated in control mice, yet not reaching significance (Supplementary Fig. S5E).

**Fig. 5:**
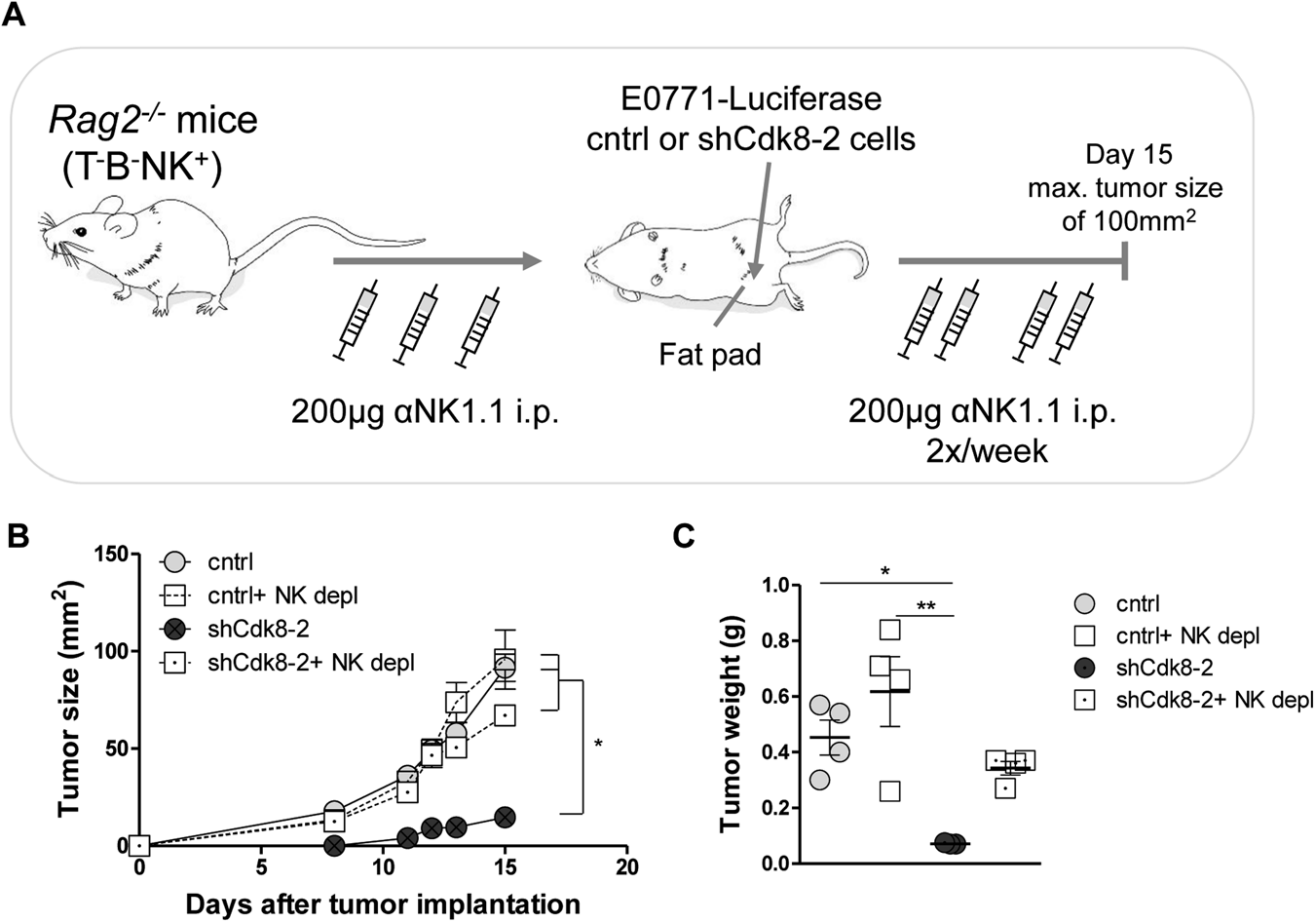
NK-cell depletion allows outgrowth of CDK8-deficient TNBC tumor cells. **A** Experimental setup of data shown in (**B**, **C**). *Rag2*^*−/−*^ mice were NK-cell depleted by i.p. injections of an αNK1.1 antibody (200 µg/mouse), three times prior to implantation of E0771 control or shCdk8-2 cells and subsequently twice per week. **B** Primary tumor growth of control vs. shCdk8-2 E0771 cells, of mice treated with αNK1.1 antibody (PBS injection served as control). Symbols and error bars represent tumor size (mm^2^) in mean ± SEM. **C** Tumor weight of mice at day 15 after tumor implantation at the endpoint of the experiment ±SEM (*n* = 4 mice per group).

To confirm that NK cells are the key players for tumor control of CDK8-deficient E0771 cells in *Rag2*^*−/−*^ mice, we performed depletion experiments using an αNK1.1 antibody. Mice were treated three times with the depletion antibody prior to tumor-cell implantation and subsequently treated twice a week (Fig. 5A). The successful depletion of CD3^−^NKp46^+^ cells was confirmed (Supplementary Fig. S5F). Again, in the control setting, CDK8-knockdown tumors grew significantly slower in *Rag2*-deficient mice. This difference was lost upon depletion of NK cells (Fig. 5B, C), unequivocally assigning the differences to NK-cell-mediated tumor surveillance.

In summary, these data define a role of tumor-cell-intrinsic CDK8 as an immune checkpoint controlling NK-cell-mediated tumor recognition. Expression of CDK8 in E0771 cells facilitates evasion from NK-cell surveillance, validating the RNA-seq-based pathway analysis.

### CDK8 contributes to IFNγ-induced PD-L1 expression

**Fig. 6:**
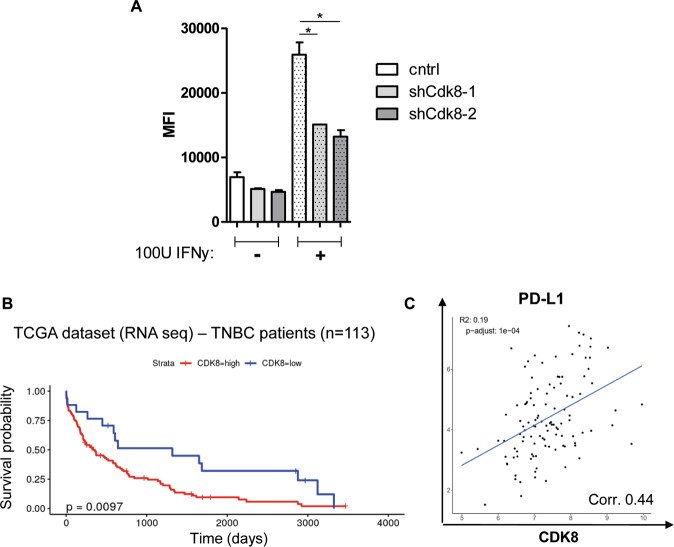
CDK8 correlates with poor prognosis and with PD-L1 expression in a human dataset. **A** Median fluorescence intensity (MFI) of PD-L1 expression of E0771 control, shCdk8-1, and shCdk8-2 cells, cultivated with or without 100 U/ml IFNγ for 48 h (indicated by “−” and “+” on the bottom of the graph); analyzed by flow cytometry. One representative experiment out of two is shown. Symbols and error bars represent mean ± SEM of technical duplicates. **B**, **C** Patient data from the TCGA database were sub-grouped in TNBC patients (*n* = 113) and analyzed for (**B**) overall survival comparing CDK8-high- and CDK8-low-expressing patients and (**C**) correlation between CDK8 and PD-L1 expression in this human dataset of TNBC patients.

The inhibitory PD-1/PD-L1 axis has recently also been implicated in the regulation of NK-cell cytotoxicity besides its important effects on cytotoxic T cells [29–31]. RNA-seq data indicated that PD-L1 (*Cd274*) expression is significantly impaired in E0771 cells upon CDK8 knockdown. We validated this finding on protein level by flow cytometry analysis (Fig. 3E). PD-L1 is regulated by interferon-γ (IFNγ) signaling and displays interferon regulatory factor 1 (IRF1) binding at its promoter, as described in melanoma cells and other cancer entities [32]. As CDK8 has been reported to act downstream of IFNγ, we stimulated E0771 cell lines with 100 U/ml IFNγ for 48 h and analyzed PD-L1 surface levels. E0771 cells significantly induced PD-L1 levels upon IFNγ treatment, which is impaired in E0771 shCdk8–1 and shCdk8–2 knockdown cells (Fig. 6A). These data suggest that regulation of PD-L1 by CDK8 might partially involve the IFNγ signaling pathway.

### CDK8 correlates with PD-L1 expression and survival in TNBC patients

To explore the human relevance of our findings, we analyzed publicly available data from large cohorts of breast cancer patients sub-filtered for TNBC patients (*n* = 113; The Cancer Genome Atlas (TCGA) RNA sequencing data). In accordance with previous studies, focusing on breast cancer patients with molecular subtypes luminal A and B, basal, and HER2^+^ [5], we extended the link of CDK8 expression to overall survival to a selected TNBC patient cohort (Fig. 6B). Analysis of correlations of CDK8 with genes identified by RNA sequencing of E0771 cells showed a significant association of CDK8 with PD-L1 in TNBC patients (Fig. 6C). These findings support the detrimental role of CDK8 in TNBC patients and link it to PD-L1 levels as potential immune evasion mechanism.

## Discussion

**Fig. 7:**
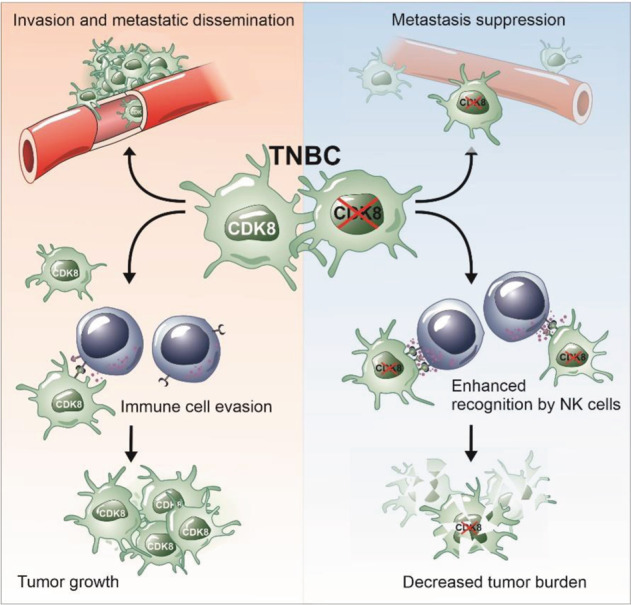
CDK8 controls metastatic properties of TNBC cells and drives NK-cell immune evasion. CDK8 in TNBC drives invasion and metastatic dissemination of tumor cells and immune evasion (left box). Loss of CDK8 in tumor cells on the one hand, dampens invasiveness and pro-metastatic behavior of tumor cells and on the other hand, enhances recognition by NK cells, thereby decreasing tumor burden (right box).

We here describe a role of CDK8 in controlling metastatic properties of TNBC cells and driving NK-cell immune evasion (Fig. 7). Whereas CDK8 is not required for cell proliferation or cell survival of TNBC, CDK8 promotes regrowth of primary tumors and subsequently the metastatic spread of TNBC cells in a murine orthotopic breast cancer model.

CDK8 has been implicated in the regulation of EMT in pancreatic, ovarian, and HER2-enriched breast cancer cell lines [20, 33] and promotes metastasis of colon cancer [34]. We confirmed the CDK8-dependent regulation of EMT markers such as *Snai2* in TNBC cells. Besides, CDK8 drives the expression of matrix metalloproteinase *Mmp9*, which contributes to the invasive character of several cancer cell types [34–36] and regulates *Ccl2* in TNBC cells, which has been found overexpressed in invasive breast cancers and is suggested to support metastasis [37–39]. Taken together, CDK8 drives a pro-metastatic program in TNBC.

Serrao et al. [20] reported reduced EMT upon CDK8/CDK19 kinase inhibition in a murine breast cancer model using Py2T cells, mimicking HER2-enriched breast cancer [40]. Our study shows opposing results as we failed to detect any deregulation of the EMT-associated transcription factors *Snai2*, as well as *Mmp9* or *Ccl2* upon CDK8/CDK19 kinase inhibition, indicating a kinase-independent regulation.

In accordance, our global RNA-seq approach identified 887 CDK8-specific genes deregulated upon CDK8 knockdown in E0771 cell lines while only 25 genes were differentially expressed upon CDK8/CDK19 kinase inhibition. This further emphasizes that in TNBC, CDK8 controls several transcriptional programs in a kinase-independent manner. The close CDK8 paralog CDK19 cannot compensate for the loss of CDK8, suggesting that CDK8 and CDK19 have non-redundant roles in TNBC. This is also evidenced by others that assign mechanistically distinct functions to CDK8 and CDK19 [41, 42].

RNA sequencing of TNBC cells also uncovered modulators of NK-cell cytotoxicity as a major CDK8-dependent pathway. Enhanced NK-cell-mediated killing of CDK8-deficient murine TNBC cells was demonstrated in vitro and confirmed in an in vivo setting using NK-cell depletion. Our experiments further demonstrate that NK cells, in principle, possess the ability to control primary tumor growth in breast cancer, but that NK-cell-mediated tumor surveillance is blocked by the presence of CDK8 in E0771 tumor cells. When CDK8 is present, breast cancer cells are not killed by NK cells and escape tumor surveillance. This might explain why several reports show that NK cells control breast cancer metastasis, but have no impact on the growth of the primary tumor [11, 13, 23, 43]. Our data now shed new light on the role of NK cells in the control of the primary tumor growth. Loss of CDK8 allows to overcome immune evasion programs that counteract NK-cell-mediated recognition of the primary tumor.

As we observed equal numbers of tumor-infiltrating NK cells in cntrl and shCdk8 tumor-bearing mice, we exclude that the enhanced surveillance is explained by a quantitative difference in NK cells. We rather hypothesize that CDK8 interferes with NK-cell-mediated tumor surveillance by regulating distinct checkpoints within TNBC cells as our transcriptome analysis revealed differential expression of crucial NK-cell-receptor ligands including the immune checkpoint protein PD-L1.

PD-L1 expression was significantly reduced on the transcriptional and translational level in E0771 cell lines lacking CDK8, identifying a new promising CDK8–PD-L1 axis in TNBC cells. PD-L1 (CD274) is a PD-1 ligand that is significantly higher expressed in TNBC compared to non-TNBC patients [2, 44]. Additionally, Phase 3 clinical trials combining chemotherapy with atezolizumab, a PD-L1 targeted antibody treatment, are promising and have given the largest overall survival benefit ever seen in patients with advanced or metastatic TNBC [45].

The consequences of PD-1 signaling for NK cells are incompletely understood, albeit there is accumulating evidence that in addition to T cells, NK cells contribute to the promising antitumor effects of PD-1/PD-L1 checkpoint inhibitors [29, 30, 46, 47]. Accordingly, we hypothesize that significantly lower levels of PD-L1 in Cdk8-knockdown cell lines are in part responsible for eliciting a stronger NK-cell-mediated antitumor response. Although a study [48] described only minimal PD-1 expression in mouse and human NK cells, we observe significant PD-1 expression on tumor-infiltrating NK cells in our model (data not shown). Our data are also supported by a positive correlation of CDK8 and PD-L1 in a dataset of human TNBC patients.

NK-cell cytotoxicity is the result of multiple influences of activating and inhibitory ligands. Thus, we believe that the reduced expression of MHC class I and NKG2D ligands, as well as the increased ICAM-1 expression contribute to the molecular mechanisms how CDK8 drives immune evasion. Low MHC class I levels point to enhanced missing self-recognition [28]. ICAM-1 is an adhesion molecule that binds LFA-1 on NK cells and contributes to the formation of the NK-cell cytotoxic immunological synapse, and thereby induces the cytotoxic activity in NK cells [49]. Although NKG2D is an activating receptor, an upregulation of NKG2D ligands has been reported to occur during EMT in colorectal cancer [50], and high levels of NKG2D ligands are associated with poor prognosis in breast cancer [51].

Comparable to the kinase-independent role of CDK8 in EMT, enhanced NK-cell-mediated recognition and elimination of E0771 cells was not recapitulated upon inhibitor treatment of the tumor cells. This also indicates a kinase-independent function of CDK8 in immune cell evasion of TNBC. Additionally, kinase-independent effects of CDK8 have also been previously described in melanoma cells and hematologic malignancies [16, 52, 53], while the effect of CDK8 on ER^+^ breast cancer progression is kinase dependent [17]. Thus, CDK8 clearly has divergent roles in different cancer entities.

In summary, we here identify CDK8 as a novel driver of immune evasion and demonstrate that targeting CDK8 in TNBC has therapeutic potential. Targeting CDK8 may be beneficial through different aspects; first, by dampening the invasive and pro-metastatic behavior of the breast cancer cells, and, second, by preventing evasion from NK cells. On top, previous studies described a suppressive effect of NK-cell-intrinsic CDK8 on NK-cell activity by regulating the lytic molecule perforin [12, 13]. Thus, targeting CDK8 in cancer patients could prevent tumor progression and immune evasion while enhancing NK-cell cytotoxicity.

As our data suggest that tumor-intrinsic CDK8 controls NK-cell immune evasion in a kinase-independent manner, CDK8/CDK19 kinase inhibitors would not suffice to reach a clinical benefit. The generation of small-molecule compounds degrading CDK8 could be a promising alternative for TNBC therapy. We believe that our data provide important mechanistic insights and pave the way for potential new options in the treatment of this fatal disease.

## Materials and methods

### Mice and cell lines

*Rag2*^*−/−*^*yc*^*−/−*^ (C.Cg-Rag2^tm1Fwa^-Il2rg^tm1Wji^) [54], *Rag2*^*−/−*^ [55], and C57BL/6J mice were in-house bred. All animals were age and gender matched (female, 9–13 weeks) for experiments and maintained at the University of Veterinary Medicine Vienna under specific pathogen-free (SPF) conditions according to Federation for Laboratory Animal Science Associations (FELASA) guidelines (2014). The animal experiments were approved by the Ethics and Animal Welfare Committee of the University of Veterinary Medicine Vienna and granted by the National Authority (Austrian Federal Ministry of Science and Research) according to Section 8ff of Law for Animal Experiments under license BMWFW-68.205/0093-WF/V/3b/2015, and were conducted according to the guidelines of FELASA and ARRIVE.

The murine TNBC cell line E0771 [56] was kindly provided by Piotr Tymoszuk (Medical University of Innsbruck) and maintained in Dulbecco’s modified Eagle’s medium (DMEM; Sigma) supplemented with 10% FCS (Bio & Sell), 100 U/ml penicillin, 100 µg/ml streptomycin (Sigma), 50 µM β-mercaptoethanol (Sigma), and 0.02 M HEPES (Sigma). Cells were cultured at 37 °C with 5% CO_2_ and regularly tested for potential *mycoplasma* contamination. E0771 cells were lentivirally infected with a CMV-Firefly Luciferase construct with ~1 × 10^7^ IFU/ml (Amsbio) and positively selected with 0.75 µg/ml puromycin (InvivoGen).

Next, E0771-Luciferase cells were transduced with CDK8-targeting shRNAs or a control shRNA (Ren.713) in a retroviral LENC (pMSCV-IRES mCherry) vector [57]. Sequences of shRNAs: Ren.713 (control): TGCTGTTGACAGTGAGCGCAGGAATTATA ATGCTTATCTATAGTGAAGCCACAGATGTATAGATAAGCATTATAATTCCTATGCCTACTGCCTCGGA; shCdk8–1: TGCTGTTGACAGTGAGCGAACAGATTATGTTAACAAAAT ATAGTGAAGCCACAGATGTATATTTTGTTAACATAATCTGTGTGCCTACTGCCTCGGA; shCdk8–2: TGCTGTTGACAGTGAGCGCTGGAGTTAGACTTGAAATGAATAGTGAAGCCACAGATGTATTCATT TCAAGTCTAACTCCAATGCCTACTGCCTCGGA.

The Phoenix ecotropic (φNX Eco) packaging system was used to produce supernatant containing retroviral pseudoparticles. To increase the virus concentration, the Retro-X^TM^ Concentrator (Clontech) was utilized according to the manufacturer’s protocol. Phoenix cells were seeded 1 day before the transfection in 10 cm dishes and grown to 50–70% confluence. Plasmid DNA was diluted in DMEM containing 10 mM HEPES buffer pH 7.4 and Turbofect^®^ Transfection Reagent (Qiagen) was added, vortexed, and incubated for 15 min at room temperature before the transfection mix was added dropwise to the cells. After harvesting and concentrating the virus supernatant, polybrene (Sigma) was added and E0771 cells were infected. Cells were sorted on a BD FACS Aria III based on mCherry expression, and CDK8 deletion was verified by western blot analysis.

### In vivo tumor models, NK-cell depletion, and imaging

To start with, 2 × 10^5^ E0771 cells were injected into the 4th mammary fat pad of female *Rag2*^*−/−*^ or *Rag2*^*−/−*^*yc*^*−/−*^ mice, and tumor growth (length and width) was measured frequently using a caliper. At a tumor area of approximately 75 mm^2^, primary tumors were surgically removed. Starting 9 days after surgery, mice were live imaged using an IVIS Lumina S5 (Perkin Elmer) system. Mice were injected with 200 µl (3 mg) D-Luciferin Bioluminescent Substrate (Perkin Elmer) i.p., narcotized with 5% isoflurane, and continuously kept in 2% isoflurane throughout the imaging procedure. At day 15 post surgery (*Rag2*^*−/−*^*yc*^−/−^ mice) or day 23 post surgery (*Rag2*^*−/−*^ mice), experiments were terminated, mice were sacrificed, imaged, and lungs were additionally imaged ex vivo. Primary tumors and lungs were minced and digested using culture media containing 1 mg/ml collagenase D (Sigma/Roche) and 20 µg/ml DNAse I (Fisher Scientific), on a gentleMACS^TM^ Octo Dissociator (Milteny Biotec). Single-cell suspensions were replated on 10 cm dishes and used for western blot or mRNA pellets.

In vivo-imaging data were analyzed using Living Image Software V 4.7.4 (Perkin Elmer).

For NK-cell depletion experiments, *Rag2*^*−/−*^ mice were injected with 200 µg αNK1.1 (PK136) antibody i.p. two times per week, starting 1 week before tumor-cell inoculation. Tumor growth was measured over time, and twice a week mice were injected with αNK1.1 antibody until termination of the experiment. Successful NK-cell depletion was verified in the blood prior to tumor-cell implantation and was confirmed at the end of the experiment.

### NK-cell isolation, expansion, and cytotoxicity assay

NK cells were isolated from spleen single-cell suspensions using DX5-labeled MACS beads according to the manufacturer’s instructions (Miltenyi Biotec). NK cells were expanded in RPMI1640 complete medium supplemented with 5000 U/ml rhIL2 (Proleukin, Novartis) for 7 days. The purity was analyzed by flow cytometry and was 85–95% CD3^−^NK1.1^+^NKp46^+^ of all living cells.

For in vitro cytotoxicity assays, 7 days expanded NK cells were mixed at indicated effector:target ratios with carboxyfluorescein diacetate succinimidyl ester (CFSE, Molecular Probes, CellTrace CFSE Cell Proliferation Kit) labeled E0771 cells. The specific target cell lysis was assessed by flow cytometry as previously described [58].

### In vitro proliferation, flow cytometry, and cell sorting

To start with, 2 × 10^4^ E0771 cells were seeded in 6-well plates in triplicate for growth curves, and counted every 2 days by flow cytometry. Analysis of NK-cell ligands was performed by staining for ICAM-1, MHC class I, RAE1, and PD-L1. Analysis of apoptotic fractions was performed by staining with AnnexinV and 7-AAD in AnnexinV 10x Staining Buffer (ThermoFisher) according to the manufacturer’s protocol. Cell cycle staining was performed using propidium iodide (Sigma).

The following antibodies (clones) were purchased from: eBioscience/Invitrogen^TM^: CD3 (17A2), NK1.1 (PK136), NKp46 (29A1.4); BioLegend^®^: CD54/ICAM-1 (YN1/1.7.4), CD274/PD-L1 (10 F.9G2); BD Pharmigen^TM^: H-2K[b] and H-2D[b] MHC class I (28-8-6); and from R&D Systems: RAE-1 (205001).

Flow cytometry experiments were performed on a BD FACSCanto II (BD Bioscience) or CytoFLEX (Beckman Coulter) and analyzed using BD FACSDiva V8.0, FlowJo V10 software and CytExpert Software V2.3.1.22. Sorting of mCherry-expressing E0771 was performed on a BD FACSAria III (BD Bioscience).

### Western blot

Whole-cell extracts were lysed in RIPA buffer (50 mM Tris-HCL (pH 7.6), 150 mM NaCl, 1% NP-40, 0.25% sodium deoxycholate, 1 mM EDTA; 20 mM β-glycero-phosphate) including cOmplete protease inhibitor cocktail (Roche) or in SDS-sample buffer. Equal amounts of proteins were separated by SDS polyacrylamide gels and transferred to a PVDF blotting membrane (GE Life science). After blocking with 5% bovine serum albumin in pY-TBST buffer (10 mM Tris/HCl pH 7.4, 75 mM NaCl, 1 mM EDTA, 0.1% Tween-20), membranes were incubated with primary antibodies overnight at 4 °C. Immunoreactive bands were visualized after incubation with the secondary antibody and Clarity Western ECL Substrate (Bio-Rad) using the ChemiDoc^TM^ Touch (Bio-Rad) and analyzed by Image Lab software (BioRad). Antibodies against CDK8 (#4106), GAPDH (#5174), pSTAT1-S727 (#9177), tSTAT1 (#9172), rabbit IgG (HRP linked, #7074 S), and mouse IgG (HRP linked, #7076 S) were purchased from Cell Signaling Technology.

### RNA Isolation and qRT-PCR

Total RNA was isolated from stable E0771 cell lines or digested primary tumors of *Rag2*^*−/−*^*yc*^*−/−*^ mice. RNA was extracted using the RNeasy Mini Kit (Qiagen) or peqGOLD TriFast (VWR) according to the protocols of the manufacturer. Reverse transcription was performed using the iScript cDNA Synthesis Kit following the manufacturer’s instructions (Bio-Rad). All quantitative PCRs (qPCRs) were performed with Sso Advanced Universal SYBR Green Supermix (Bio-Rad) according to the instructions of the manufacturer. Expression of mRNA was normalized to *Rplp0* mRNA. The following primers were used: *Rplp0* fwd: GCTTTCTGGAGGGTGTCC, rev: GCTTCAGCTTTGGCAGGG; *Snai2* fwd: TGTGTCTGCAAGATCTGTGGC, rev: TCCCCAGTGTGAGTTCTAATGTG; *Mmp9* fwd: TGTCTGGAGATTCGACTTGAAG, rev: ATAGGCTTTGTCTTGGTACTGG; *Ccl2* fwd: TCAGCCAGATGCAGTTAACGCCC, rev: GCTTCTTTGGGACACCTGCTGCT.

### RNA sequencing analysis

Libraries from untreated murine E0771 control or Senexin B (SnxB)-treated cells, and shCdk8–1 or shCdk8–2 cell lines in triplicate were prepared using the NEBNext Multiplex Oligos for Illumina (Dual Index Primers). Single-end, 50 bp sequencing was performed on an Illumina HiSeqV4 SR50 sequencer (Illumina, San Diego, CA, USA). After quality control of raw data with FastQC and removal of adapters and low-quality reads with Trimmomatic (version 0.36), reads were mapped to the GENECODE M13 genome using STAR (version 2.5.2b) with default parameters. Counts for union gene models were obtained using featureCounts from the Subread package (version 1.5.1). Differentially expressed (Benjamin–Hochbert corrected *p*-value (*p*-adjust) < 0.05 and fold change >2) genes were identified using edgeR (version 3.30.3). Over-representation analysis was done using the STRING enrichment API method (https://string-db.org/cgi/help.pl?subpage=api%23getting-functional-enrichment). The RNA-seq data reported in this article have been deposited in the Gene Expression Omnibus database (Accession ID: GSE161295).

### Inhibitors

E0771 cells were incubated for 48 h with the CDK8/CDK19 inhibitor Senexin B (Gentaur GmBH/APExBio) or DMSO as a control. Inhibitor concentrations were first titrated based on pSTAT1-S727 levels using western blotting. For NK-cell cytotoxicity assays, 1 µM Senexin B was applied and kept in the medium throughout the assay. Treatment with 1500 U/ml IFNγ (Merck) for 30 min was used to induce STAT1-S727 phosphorylation. For experiments analyzing surface expression of PD-L1 by flow cytometry, E0771 cells were treated with 100 U/ml IFNγ for 48 h.

### Human patient data

RNA-seq data from TNBC patients from TCGA was obtained from the R package MetaGxBreast by selecting for samples with an estrogen receptor, progesterone receptor, and HER2-negative status. Differences in overall survival between groups were calculated using the log-rank test and visualized by Kaplan-Meier curves using the ggsurvplot function from the same package. Pairwise correlations (Spearman) between the expression levels of CDK8 and genes found significantly regulated in CDK8-knockdown E0771 breast cancer cell lines (relative to control) of TNBC patients, and were calculated using the corr.test function from base R. Correction for multiple testing was done with the base R function p.adjust (method “BH”). Coefficient of determination (=R2) and Benjamini Hochberg corrected *p*-value are indicated in the plots. Values in the plots represent mRNA expression *z*-scores.

### Statistical analysis

Kruskal–Wallis test (followed by Dunn’s test), one-way analysis of variance (followed by Tukey’s multiple comparison test), unpaired *t*-test, Mann–Whitney test and a linear regression model were performed with log transformed data using GraphPad Prism^®^ Software version 5.04 and R Studio version 1.2.1335. All data are shown as mean ± SEM. Statistical significance is indicated for each experiment (*p < 0.05, **p < 0.01, ***p < 0.001, ****p < 0.0001).

## Supplementary information

Supplementary information.

## Data Availability

The data that support the findings of this study are available from the corresponding author upon request. The RNA-seq data reported in this article are available in the Gene Expression Omnibus database under accession number GSE161295.
