# Supplementary material for: Triple-negative breast cancer cells rely on kinase-independent functions of CDK8 to evade NK-cell-mediated tumor surveillance
Source: Cell Death Dis. 2021 Oct 23;12(11):991. doi: 10.1038/s41419-021-04279-2 (PMC8542046; doi:10.1038/s41419-021-04279-2)
Supplement: Supplementary file 1 — Supplementary information. [file 41419_2021_4279_MOESM1_ESM.pdf]

## Supplementary Figures

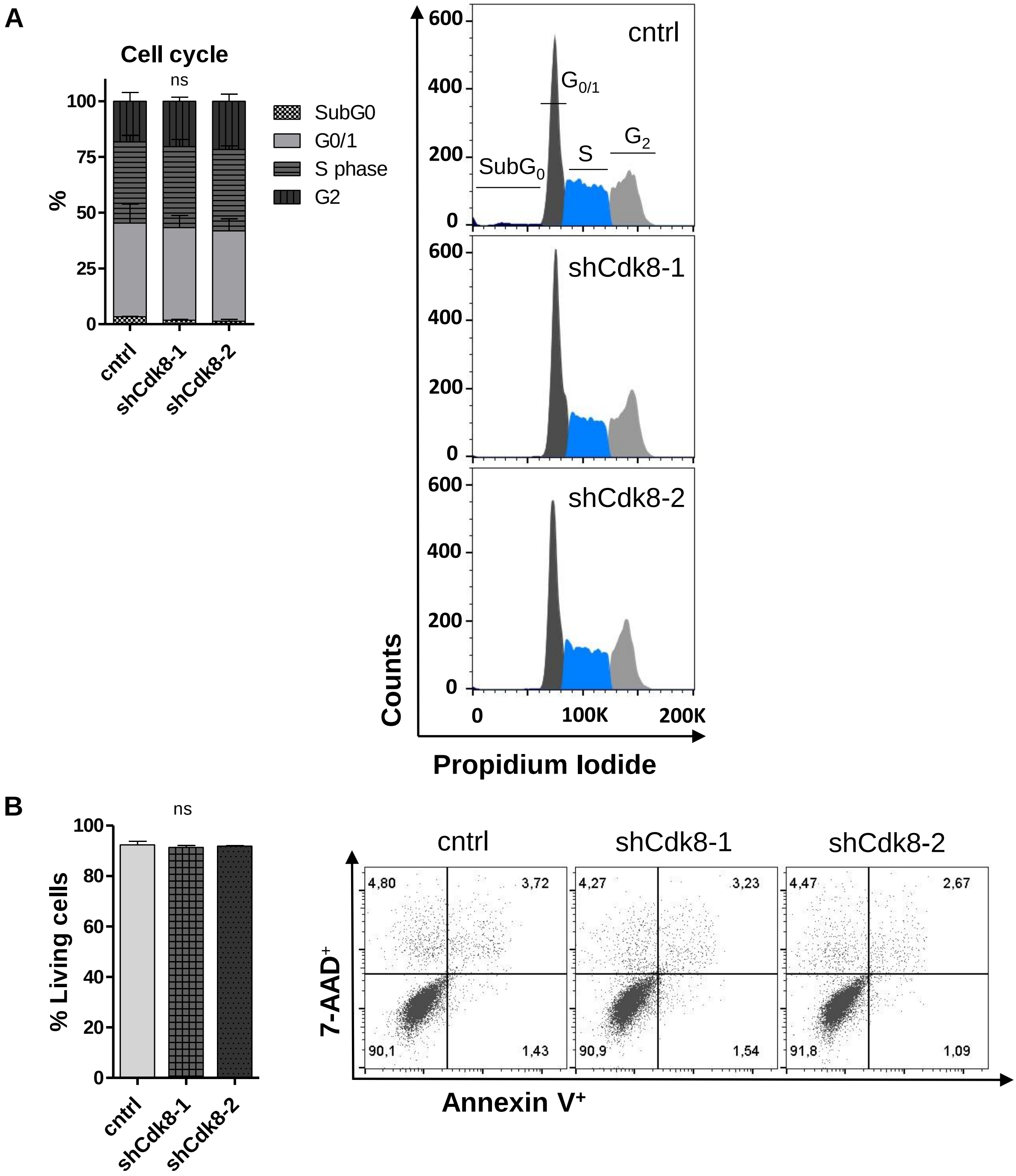


**Supplementary Figure S1. Loss of CDK8 does not alter survival or cell cycle progression of murine TNBC cells.**

(**A**) PI cell cycle staining and (**B**) Annexin V/ 7-AAD staining of control, shCdk8-1 and shCdk8-2 E0771 cells. Graphs show mean ± SEM of two pooled independent experiments. Representative histograms (**A**) and FACS blots (**B**) are shown.

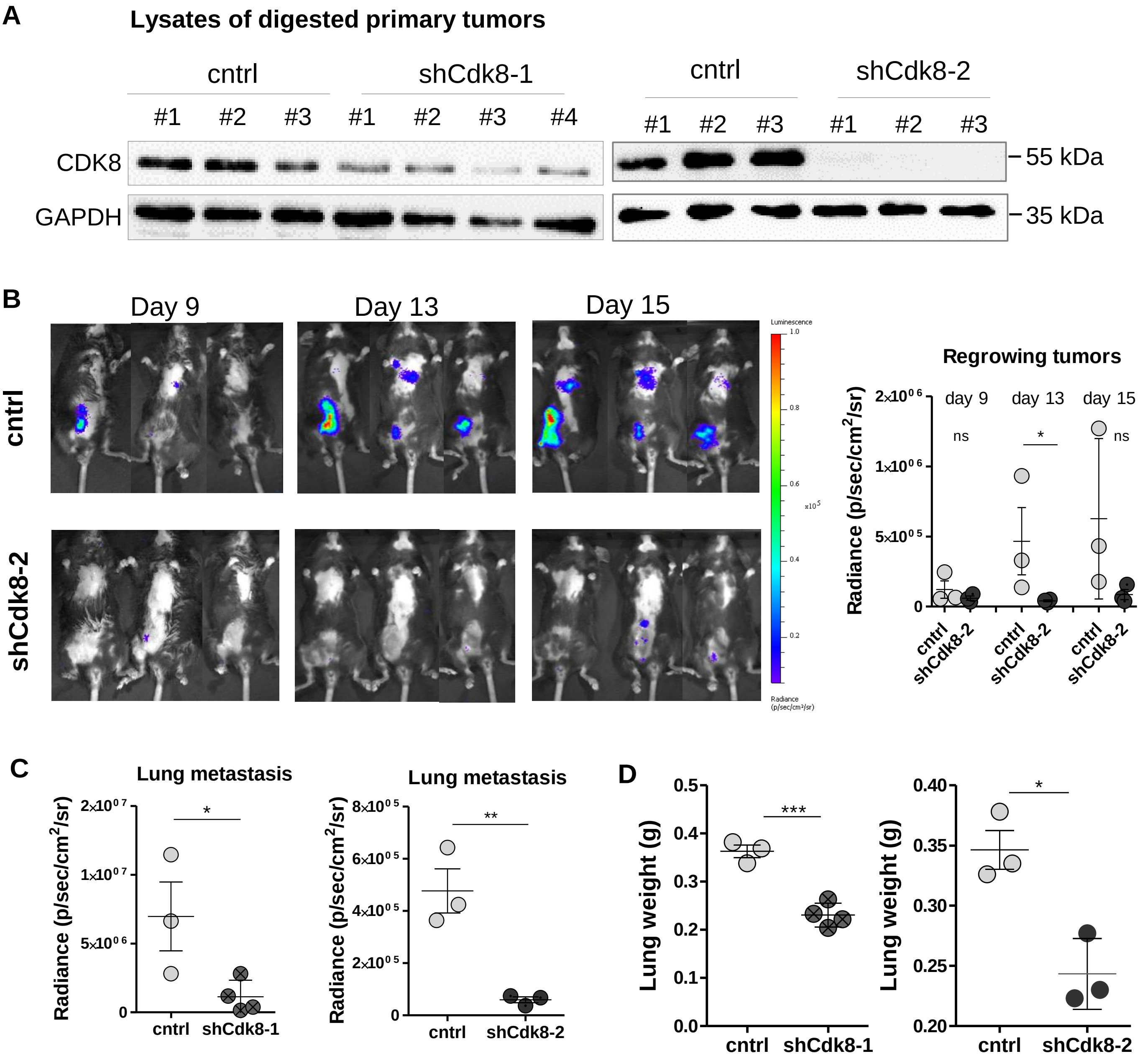


**Supplementary Figure S2. CDK8 supports the re-growth of tumors and distant metastasis formation.**

(**A**) Lysates of digested primary tumors were analyzed for CDK8 expression by western blotting. GAPDH was used as a loading control. (**B**) Representative *in vivo* imaging pictures of mice (left) and evaluation of measured radiance signals ± SEM (right) of mice implanted with E0771 control versus shCdk8-2 cells. *In vivo* imaging was performed on day 9, day 13 and day 15 post-surgery (endpoint of the experiment). (**C**) Radiance signals of *ex vivo* lung metastasis and (**D**) lung weight ± SEM of two independent experiments, at day 15 post-surgery.

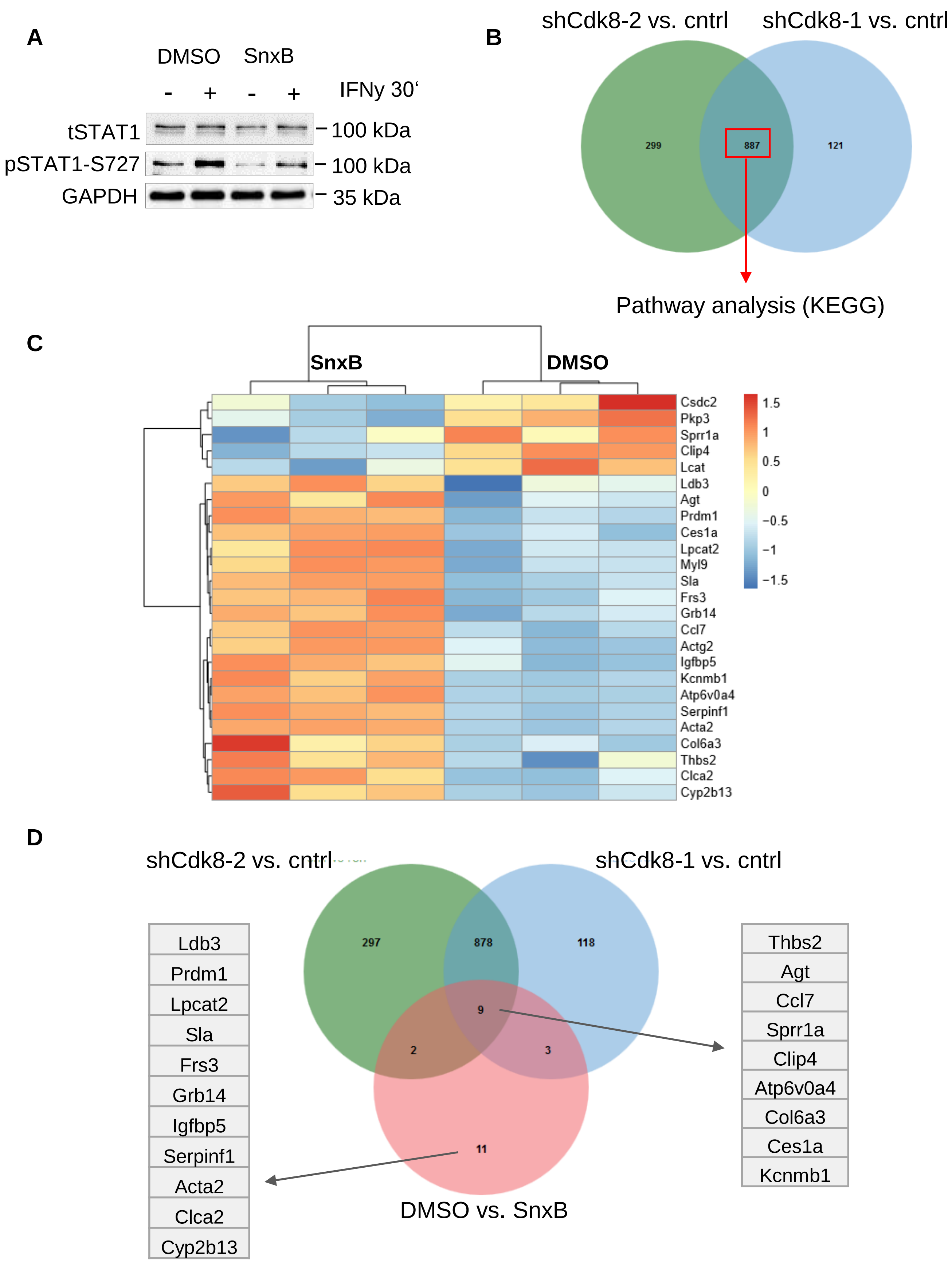
A
DMSO
SnxB
- + - +
IFNy 30‘
tSTAT1
pSTAT1-S727
GAPDH
100 kDa
100 kDa
35 kDa
B
shCdk8-2 vs. cntrl
shCdk8-1 vs. cntrl
299
887
121
Pathway analysis (KEGG)
C
SnxB
DMSO
Csdc2
Pkp3
Sprr1a
Clip4
Lcat
Ldb3
Agt
Prdm1
Ces1a
Lpcat2
Myl9
Sla
Frs3
Grb14
Ccl7
Actg2
Igfbp5
Kcnmb1
Atp6v0a4
Serpinf1
Acta2
Col6a3
Thbs2
Clca2
Cyp2b13
1.5
1
0.5
0
−0.5
−1
−1.5
D
shCdk8-2 vs. cntrl
shCdk8-1 vs. cntrl
297
878
118
9
2
3
11
DMSO vs. SnxB
Ldb3
Prdm1
Lpcat2
Sla
Frs3
Grb14
Igfbp5
Serpinf1
Acta2
Clca2
Cyp2b13
Thbs2
Agt
Ccl7
Sprr1a
Clip4
Atp6v0a4
Col6a3
Ces1a
Kcnmb1

**Supplementary Figure S3. CDK8/CDK19 inhibitor treatment does not mimic genetic knockdowns of CDK8.**

(**A**) E0771 control cells were treated with 1µM Senexin B (SnxB) or DMSO for 48 hours and incubated ± IFNy for 30min and blotted for STAT1, pSTAT1-S727 and GAPDH expression. (**B**) Venn diagram depicting overlap of genes regulated by two different CDK8-targeting shRNAs (shCdk8-1 and -2). (**C**) Heatmap of 25 differentially expressed genes between E0771 control cells treated with 1µM Senexin B (SnxB) or DMSO for 48 hours (RNA-seq data; n=3 replicates). (**D**) Venn diagram depicting overlaps of genes regulated upon Senexin B treatment with genes regulated by two different CDK8-targeting shRNAs (shCdk8-1 and -2). Left table shows 11 genes solely regulated by Senexin B treatment and right table depicts 9 genes regulated upon Senexin B treatment and genetic CDK8 knockdowns.

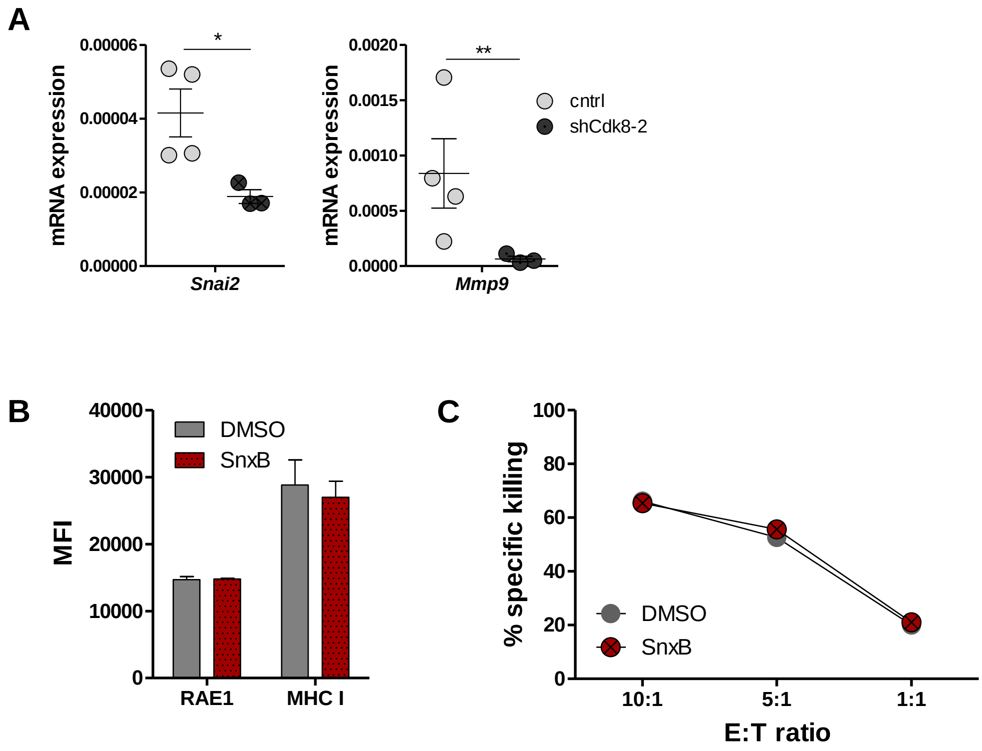


**Supplementary Figure S4. CDK8/CDK19 inhibitor treatment of E0771 does not alter their killing by NK cells.**

(**A**) Real-time quantitative PCR of RNA extracted from digested primary tumors of control versus shCdk8-2 implanted *Rag2*$^{-/-}$*γc*$^{-/-}$ mice ± SEM. Expression levels of *Snai2* and *Mmp9* were normalized to the house keeping gene *Rplp0*. (**B**) MFI of RAE1 and MHC I (H-2K+ H-2D) expression of E0771 cells treated with 1µM Senexin B (SnxB) or DMSO as a cntrl; expression levels of NK ligands were analyzed by flow cytometry. Bar graphs represent technical triplicates (mean ± SEM). (**C**) IL2-expanded C57BL/6 NK cells were incubated for 4 hours with CFSE-labelled E0771 control tumor cells pretreated with 1µM Senexin B (SnxB) or DMSO for 44 hours in effector:target (E:T) ratio of 10:1, 5:1 and 1:1. The specific killing was assessed by flow cytometry. One representative experiment out of two independent experiments is shown.

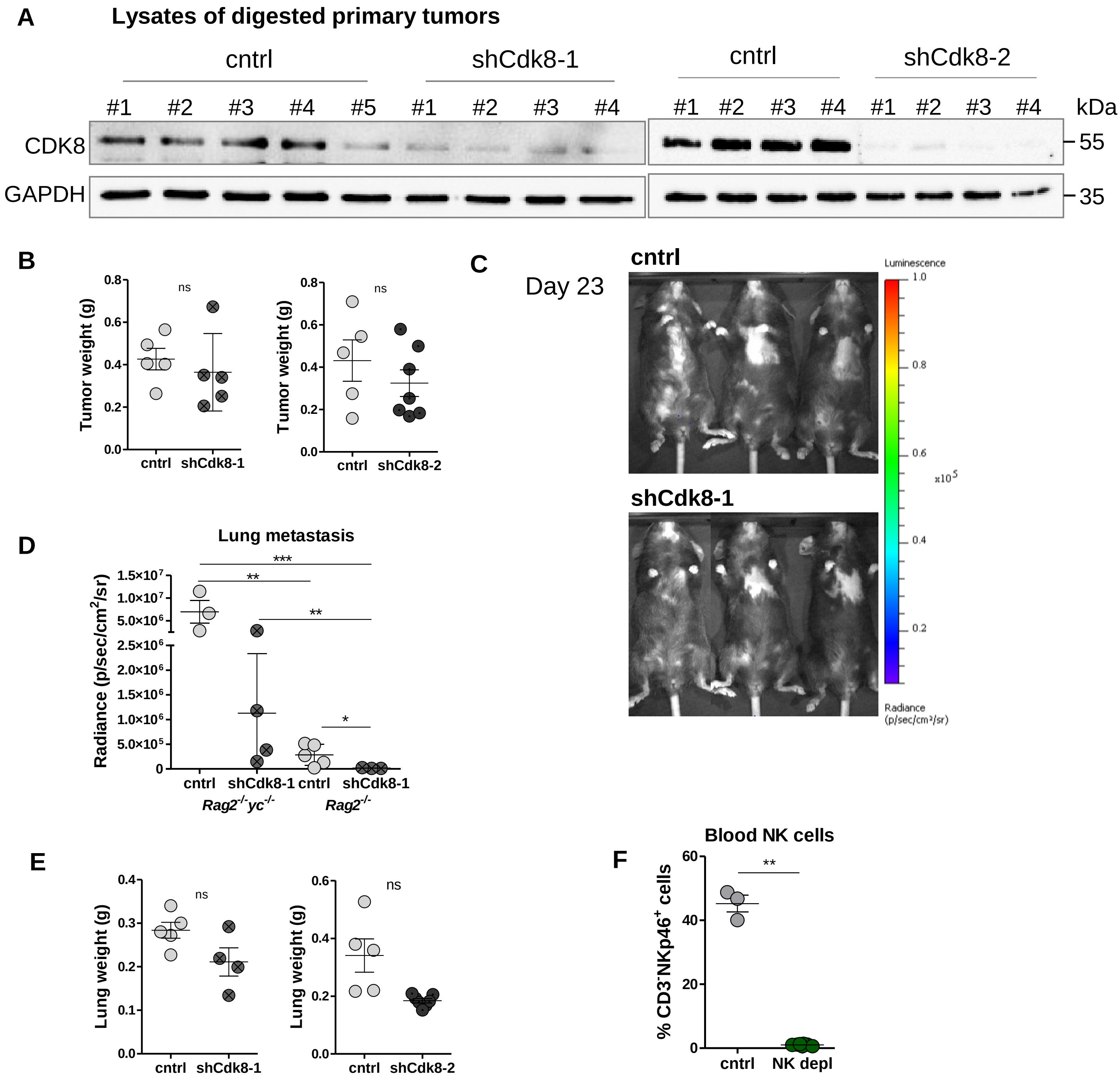


**Supplementary Figure S5. Loss of CDK8 in TNBC increases NK-cell mediated tumor surveillance.**

(**A**) Lysates of digested primary tumors were analyzed for CDK8 expression by western blotting. GAPDH was used as a loading control. (**B**) Tumor weight (g) ± SEM of *Rag2*$^{-/-}$ mice shown in Figure 4B. (**C**) Representative *in vivo* imaging pictures of *Rag2*$^{-/-}$ mice on day 23 post surgery. (**D**) Comparison of radiance signals of lung metastasis ± SEM of *Rag2*$^{-/-}$*γc*$^{-/-}$ (on day 15 post-surgery) and *Rag2*$^{-/-}$ mice (on day 23 post-surgery) implanted with control versus shCdk8-1 TNBC cells. (**E**) Lung weight (g) of *Rag2*$^{-/-}$ mice ± SEM. (**F**) Percentage of $CD3^{-}NKp46^{+}$ NK cells ± SEM in blood of control and NK-cell depleted mice, after three injections of αNK1.1 antibody and prior to tumor cell implantation; percentage of NK cell numbers were analyzed by flow cytometry.
